# Identification of fungal diseases in strawberry by analysis of hyperspectral images using machine learning methods

**DOI:** 10.18699/vjgb-25-34

**Published:** 2025-04

**Authors:** A.F. Cheshkova

**Affiliations:** Siberian Federal Scientific Centre of Agro-BioTechnologies of the Russian Academy of Sciences, Krasnoobsk, Novosibirsk region, Russia

**Keywords:** hyperspectral imaging, fungal diseases of strawberries, machine learning methods, dimensionality reduction, гиперспектральные изображения, грибные болезни земляники, методы машинного обучения, сокращение размерности

## Abstract

Leaf spot, leaf scorch and phomopsis leaf blight are the most common fungal diseases of strawberry in Western Siberia, which significantly reduce its yield and quality. Accurate, fast and non-invasive diagnosis of these diseases is important for strawberry production. This article explores the ability of hyperspectral imaging to detect and differentiate symptoms caused to strawberry leaves by pathogenic fungi Ramularia tulasnei Sacc., Marssonina potentillae Desm. and Dendrophoma obscurans Anders. The reflection spectrum of leaves was acquired with a Photonfocus MV1-D2048x1088-HS05-96-G2-10 hyperspectral camera under laboratory conditions using the line scanning method. Five machine learning methods were considered to differentiate between healthy and diseased leaf areas: Support Vector Machine (SVM), K-Nearest Neighbors (KNN), Linear Discriminant Analysis (LDA), Partial Least Squares Discriminant Analysis (PLS-DA), and Random Forest (RF). In order to reduce the high dimensionality of the extracted spectral data and to increase the speed of their processing, several subsets of optimal wavelengths were selected. The following dimensionality reduction methods were explored: ROC curve analysis method, derivative analysis method, PLS-DA method, and ReliefF method. In addition, 16 vegetation indices were used as features. The support vector machine method demonstrated the highest classification accuracy of 89.9 % on the full range spectral data. When using vegetation indices and optimal wavelengths, the overall classification accuracy of all methods decreased slightly compared to the classification on the full range spectral data. The results of the study confirm the potential of using hyperspectral imaging methods in combination with machine learning for differentiating fungal diseases of strawberries.

## Abstract (ru)

Белая, бурая и угловатая пятнистости являются наиболее распространенными грибными болезнями земляники садовой в Западной Сибири, значительно влияющими на ее урожайность и качество. Точная, быстрая и неинвазивная диагностика этих заболеваний имеет важное значение в промышленном производстве земляники. В настоящей статье исследуются возможности применения методов машинного обучения и гиперспектральной визуализации для обнаружения и дифференциации на листьях земляники симптомов, вызванных патогенными грибами Ramularia tulasnei Sacc., Marssonina potentillae Desm. и Dendrophoma obscurans Anders. Спектр отражения листьев регистрировали гиперспектральной камерой Photonfocus MV1-D2048x1088-HS05-96-G2-10 в лабораторных условиях методом линейного сканирования. Для дифференциации здоровых и пораженных областей листьев изучено пять методов машинного обучения: метод опорных векторов (SVM), метод К-ближайших соседей (KNN), линейный дискриминантный анализ (LDA), дискриминантный анализ частичных наименьших квадратов (PLS-DA) и случайный лес (RF). С целью уменьшения высокой размерности извлеченных спектральных данных и увеличения скорости их обработки было отобрано несколько подмножеств оптимальных длин волн, несущих наиболее важную спектральную информацию. Рассмотрены следующие методы сокращения размерности: метод анализа ROC-кривых, метод анализа производных, метод PLS-DA, метод ReliefF. Кроме того, 16 вегетационных индексов задействовано в качестве информативных признаков. Наибольшую точность классификации, 89.9 %, показал метод опорных векторов на полном спектре значений. При ис- пользовании вегетационных индексов и наборов оптимальных длин волн общая точность классификации всех методов снизилась незначительно по сравнению с классификацией на полном спектре значений. Результаты исследования подтверждают перспективность применения методов гиперспектральной визуализации в сочетании с методами машинного обучения для дифференциации грибных болезней земляники садовой

## Introduction

Strawberry is one of the most popular fruits among consumers by flavor, nutritional value and health benefits (Zheng et al., 2021). Strawberry has high productivity and profitability and is capable of rapid vegetative reproduction. One of the limiting factors for increasing the production of strawberries is the significant damage to cultivated varieties by fungal diseases, which leads to a decrease in yield and economic losses. The most common fungal diseases of strawberries in Western Siberia are leaf spot, leaf scorch and phomopsis leaf blight (Govorova, Govorov, 2015). Early detection of these diseases is crucial for targeted application of appropriate plant protection measures

Traditional disease diagnostic methods such as visual assessment and microbiological laboratory analysis are timeconsuming, error-prone and labor-intensive, which limits their application in precision agriculture. Recently, hyperspectral image analysis (Mishra et al., 2017; Mahlein et al., 2018; Cheshkova, 2022) has demonstrated great potential as an effective and non-invasive method for monitoring plant biotic and abiotic stress. The influence of pathogens causes changes of the physiological and biochemical parameters in the process of disease occurrence, creating a reflectance spectrum that is different from the spectrum of healthy plants. Modern optical sensors register up to several hundred bands of the electromagnetic spectrum over a wide range of wavelengths and form a spectral profile for each pixel combining spectral and spatial information (Mishra et al., 2017). Hyperspectral imaging combines the advantages of computer vision and optical spectroscopy, allowing simultaneous assessment of both physiological and morphological parameters. Currently, scientific publications provide examples of the successful use of hyperspectral imaging for the recognition of various strawberry diseases, such as powdery mildew (Mahmud et al., 2020), anthracnose (Lu et al., 2017; Jiang et al., 2021), verticillium wilt (Cockerton et al., 2019), gray mold (Wu et al., 2023), and spotting (Cheshkova, 2023).

Machine learning is one of the most effective ways to process and analyze the vast amounts of data obtained by remote sensing techniques (Nagaraju et al., 2020; Benos et al., 2021). Numerous studies show that using vegetation indices as features for building machine learning models allows achieving good results in identifying and recognizing diseases of agricultural crops (Mahlein, 2013; Lu et al., 2017).

Hyperspectral data has high collinearity. A large number of wavelengths complicates models and reduces performance. Dimension reduction is specific and significant for hyperspectral- based plant disease analysis, the purpose of which is to remove spectral redundancy while preserving important information. Optimal waveband selection has always been a primary concern in hyperspectral data analysis (Liu et al., 2014; Sun, Du, 2019). Уменьшение размерности может быть достигнуто за счет выбора определенных длин волн либо выделения информативных признаков

The objective of this study was to determine the efficiency of hyperspectral imaging techniques for differentiating symptoms on strawberry leaves caused by pathogenic fungi Ramularia Tulasnei Sacc., Marssonina potentillae Desm. and Dendrophoma obscurans Anders.; to assess the accuracy of different machine learning methods for identifying fungal diseases of strawberry; to explore the possibility of using dimensionality reduction methods and vegetation indices to optimize the machine learning models.

## Materials and methods

Plant material and fungal diseases. In our study, three types of fungal diseases of strawberry, most common in Western Siberia, were considered: leaf spot, leaf scorch and phomopsis leaf blight.

Strawberry leafspot is caused by Mycosphaerella fragariae (Tul.) Lindau (conidial stage: Ramularia Tulasnei Sacc.). The disease is first noticed as small, purplish circular spots on the surface of young leaflets. As the lesion enlarges, the center of the spot becomes gray to white and is surrounded by distinct reddish-brown borders.

Strawberry leaf scorch is caused by Diplocarpon earliana (Ell. et Ev.) Wolf (conidial stage: Marssonina potentillae (Desm.) Р. Magn., M. fragariae (Lib.) Ohl.). The marks of the disease consist of many small irregular purple spots that appear on the outward leaf’s surface. The lesions may enlarge to 5 mm across and appear irregular.

Phomopsis leaf blight is caused by Dendrophoma obscurans (Ell. et Ev.) H.W. Anderson (synonym: Phomopsis obscurans (Ell. et Ev.) Sutton). Lesions begin as circular to elliptical, purple spots that can appear identical to those of common leaf spot or leaf scorch. The purple spots develop dark brown centers as they enlarge. Some infected leaves display large V-shaped lesions, with the widest part at the leaf edge.

The strawberry plants grown at the experimental field of Siberian Federal Scientific Centre of Agro-BioTechnologies of the Russian Academy of Sciences (Krasnoobsk, Novosibirsk region, Russia) in 2021–2023 were used in the overall experiment. During the growing seasons, 120 plants were selected, including 30 healthy plants and 30 plants for each disease: with visible symptoms of leaf spot, leaf scorch or phomopsis leaf blight. One leaf from each plant was detached for further research in the laboratory. Identification of the disease was carried out through visual expertise by symptoms of the disease (Garrido et al., 2011; Govorova, Govorov, 2015).

Image acquisition and calibration. Imaging was performed by a Photonfocus MV1-D2048x1088-HS05-96-G2-10 hyperspectral camera, with an IMEC CMV2K-LS150-VNIR sensor (Photonfocus AG, Switzerland, wavelength range 470–900 nm, spectral resolution 3 nm, spatial resolution 2048 × 1088 pixels) by the linear scanning method using a moving platform. The software and hardware equipment as well as related parameters can be referred to article (Maximov et al., 2023). The strawberry leaves were placed on a white platform for imaging. The illumination source was two halogen lamps. The scanning step, exposure time and camera mounting height were determined experimentally. Additionally, reference images were created for radiometric correction. The dark image was obtained by covering the camera lens with a non-reflective opaque black cap, and the white reference image was obtained by surveying the spectral image of the Teflon white board with 99.9 % reflectance. The calibrated image was calculated using the following formula where IS – intensity value of the sample image, ID – intensity value of the dark reference image, IW – intensity value of the white reference image, R – the corrected hyperspectral reflectance image.

**Formula. 1.**
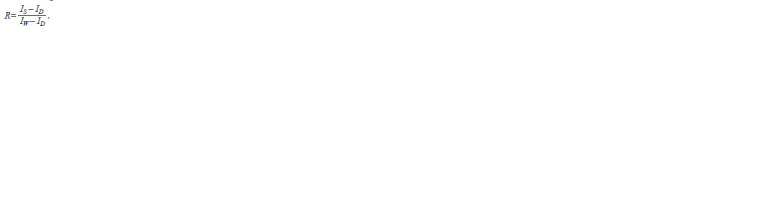
Formula. 1.

Three-dimensional data sets (hypercubes) containing two dimensions of spatial information and additionally one dimension of spectral information (2048 × 1088 × 131) were formed from the scanning results

**Fig. 1.**
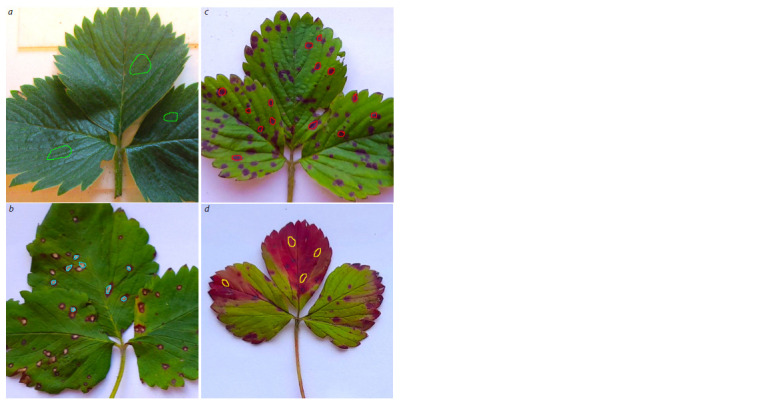
ВROI selection: a – healthy regions, b – regions with symptoms of leaf spot, c – regions with symptoms of leaf scorch, d – regions with symptoms of phomopsis leaf blight.

Spectral features extraction and processing. The resulting image files were divided into two groups: 96 leaf images to form a training set (24 in each of the four classes) and 24 leaf images to form a validation set (six in each of the four classes). Spectral data extraction was performed using ENVI 5.2 (NV5 Geospatial Solutions, Inc., USA). In each strawberry leaf image, regions of interest (ROIs) corresponding to healthy leaf tissue and to color spots of the diseased tissue were manually selected (Fig. 1). From each region, 250 pixels were randomly selected. As a result, a training dataset of 24,000 spectrum values (6,000 px for each class) and a validation dataset of 6,000 spectrum values (1,500 px for each class) were formed.

To smooth the spectrum and correct for scattering, the Savitzky–Golay filter (Savitzky, Golay, 1964) and standard normal variate normalization (Vidal, Amigo, 2012) were applied to the spectral data

Optimal wavelengths selection. In order to decrease the dimension of the raw spectral information and to find the optimal wavelengths for classification we examined the following dimensionality reduction methods:

– receiver operating characteristic (ROC) analysis (Luo et al., 2012); in this method, the area under curve (AUC) value is used as a metric that determines the variable importance; from the entire data spectrum, those wavelengths are left for which the AUC exceeds a certain threshold value; – the derivative analysis (Savitzky, Golay, 1964); in this method, the most important wavelengths are selected as the high peaks and low valleys in the second derivative plot; – partial least squares discriminant analysis (PLS-DA) (Mehmood et al., 2012); in this method, the wavelengths that correspond to the highest absolute values of β-coefficients are considered optimal wavelengths; – ReliefF algorithm (Kononenko, 1994; Urbanowicz et al., 2018); it’s a feature weighting algorithms that assigns different weights to features based on the category and correlation of each feature; features with weights below a certain threshold value are removed.

Vegetation indices extraction. Vegetation indices are algebraic combinations calculated from reflectance spectrum values for two or more selected wavelengths.

For our study, 16 vegetation indices (Table 1) were selected, characterizing the photochemical reflectance (PRI), physiology (NDVI, RENDVI, RVSI, PhRI), content of chlorophyll (MCARI, TVI, VOG1, VOG2, VOG3), pigments (PSSRa, PSSRb, CRI, ARI), nitrogen (NRI) and carbon (PSRI) in plant leaves (Wu et al., 2023).

**Table 1.**
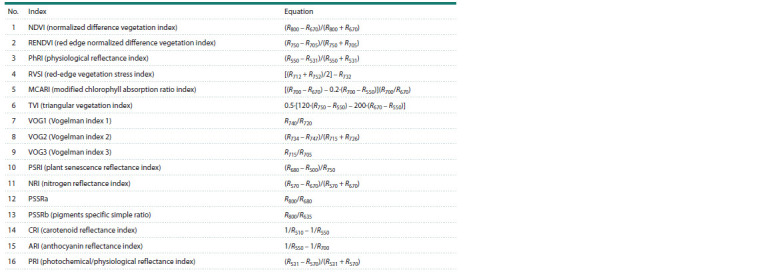
Vegetation indices used as features

Machine learning methods. Five machine learning methods (SVM, KNN, LDA, PLS-DA, RF), most commonly used in hyperspectral data classification (Singh et al., 2016; Benos et al., 2021), were considered in our study to differentiate healthy and diseased regions of strawberry leaves.

The Support Vector Machine (SVM) method. The main idea of the SVM method is to transfer the original vectors to a higher-dimensional space and search for the separating hyperplane with the largest gap in this space. The Gaussian radial basis function was taken as the classifier kernel.

The K-Nearest Neighbors (KNN) method. The classification is achieved by assigning the test object to the class that is most common among its K-nearest neighbors, the classes of which are already known. It applies the Euclidean distance in the multidimensional space as a similarity measurement to separate the test objects.

The Linear Discriminant Analysis (LDA) method. The high-dimensional data are projected into a lower-dimensional space to promote class separability. The optimal projection in classical LDA is obtained by maximizing the distance between different classes and minimizing the distance within a class

The Partial Least Squares Discriminant Analysis (PLS-DA) method. It is a variant of combining Partial Least Squares regression (PLSR) and discriminant analysis (DA). Unlike classical discriminant analysis, which searches for hyperplanes of maximum variance of independent predictors, PLS-DA builds a linear regression model by projecting predicted and observed variables into a new reduced space.

The Random Forest (RF) method. It is a non-parametric method that uses multiple decision trees to classify data and is well suited to spectral data analysis.

The overall accuracy, calculated as the percentage of correctly classified objects to the total number of objects, was used as a metric to evaluate the quality of the models.

All calculations and data analysis were performed in the R software using the caret, kernlab, randomForest, klaR, pls, CORElearn, class, MASS and terra packages.

## Results

Spectral behaviors

**Fig. 2.**
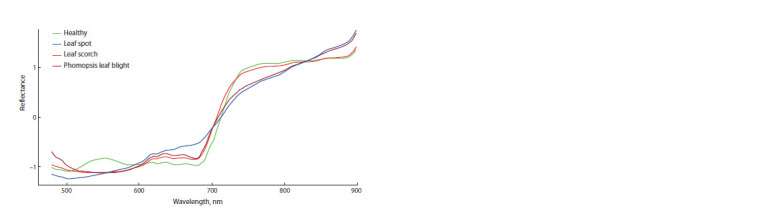
Average reflectance spectrum of healthy and infected regions of strawberry leaves.

Figure 2 shows the averaged reflectance spectra of healthy and fungal disease-affected regions of strawberry leaves. The spectral curves are typical for plants (Mishra et al., 2017). A common feature of all spectral curves is a lower reflectance in the visible wavelength range, compared to the near-infrared range. At wavelengths around 670 nm, a decrease in reflectance is observed, which is due to the strong absorption of light by chlorophyll in the leaves. In the range of 670–760 nm, the reflectance of leaves increases sharply due to light scattering in the intercellular space. In the wavelength range of 760–900 nm, the reflectance remains high

Certain differences between the spectra are observed. Thus, healthy green leaf regions have a characteristic peak at a wavelength of 550 nm (nitrogen absorption zone), while diseased regions have a decline in this area. In the range of 720–810 nm, healthy regions and regions affected by leaf scorch have a higher reflectance, compared to regions affected by leaf spot and phomopsis leaf blight. And in the range of 810–900 nm, on the contrary, it is lower. The reflectance of leaves affected by leaf spot disease increases uniformly over the entire wavelength range.

Optimal wavelengths selection

Analysis of variance (ANOVA) revealed the statistically significant differences between mean reflectance by disease type for each of the wavelengths, according to the F-criterion with a p-value < 0.001. In addition, a recursive feature elimination method was applied to each of the considered models, which also revealed that all wavelengths were significant for classification.

To reduce the dimensionality of the data, four different techniques were considered, which determined four different sets of optimal wavelengths (Figures S1–S4 in Supplementary Materials)1.


Supplementary Materials are available in the online version of the paper: https://vavilov.elpub.ru/jour/manager/files/Suppl_Cheshkova_Engl_29_2.pdf


Using ROC curve analysis, 23 wavelengths (nm) were identified for which the AUC exceeded the threshold value of 0.99: [541.39, 545.04, 548.92, 550.41, 553.99, 557.94, 561.3, 565.18, 568.58, 745.48, 748.98, 751.75, 756.45, 759.36, 763.0, 765.97, 769.44, 772.39, 775.92, 778.56, 781.11, 784.53, 787.2].

Using the second derivative analysis method with a threshold value of 1.0, the following 15 wavelengths (nm) were selected: [677.11, 680.47, 682.99, 685.28, 688.76, 691.62, 695.25, 697.97, 709.54, 712.19, 729.07, 732.25, 736.15, 739.20, 742.67].

Using the PLS-DA method, the following 16 wavelengths (nm) were selected for a threshold value of regression coefficients of 0.4: [498.68, 502.7, 505.97, 510.11, 513.5, 517.33, 522.39, 526.49, 529.98, 533.99, 541.39, 680.47, 682.99, 688.76, 691.62, 722.02].

Using the ReliefF method, the following 24 wavelengths (nm) were selected for the weight threshold of 0.5: [537.27, 541.39, 545.04, 548.92, 550.41, 557.94, 561.3, 565.18, 657.08, 662.06, 664.83, 668.35, 670.93, 674.46, 677.11, 680.47, 682.99, 685.28, 688.76, 691.62, 751.75, 756.45, 759.36, 763.00].

**Fig. 3.**
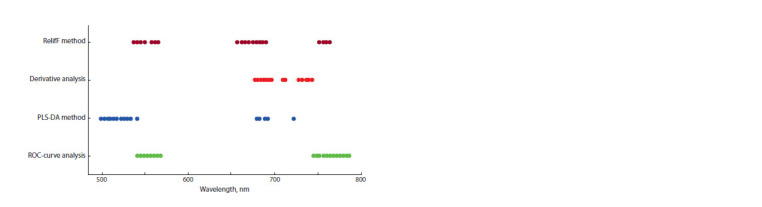
Sets of optimal wavelengths determined by different methods.

A comparison of the sets of optimal wavelengths selected by different methods (Fig. 3) allows us to conclude that the most informative wavelength ranges for classification are [542–565 nm] and [680–691 nm].

Vegetation indices calculation and analysis

Sixteen vegetation indices were calculated using the corresponding formulas (Table 1) for each pixel in the dataset. Analysis of variance (ANOVA) was performed for each index to determine the statistical significance of differences between mean values of indices by disease type. All 16 indices had statistically significant differences between means with a p-value < 0.001.

Classification results based on the full range of wavelengths

In our study, five different models (SVM, KNN, LDA, PLSDA, RF) were applied to differentiate healthy and fungal disease-affected strawberry leaves. First, models were built for the full spectrum of wavelengths (131 wavelengths in the range 470–900 nm). The following optimal hyperparameters were selected using the cross-validation: SVM (sigma = 0.03, C = 6), KNN (K = 9), RF (mtry = 11), PLS-DA (ncomp = 38). The classification results are shown in Table 2. Analysis of the results allows us to conclude that the main errors in classification occur when differentiating between leaf scorch and phomopsis leaf blight, since these areas have a similar reflectance spectrum.

**Table 2.**
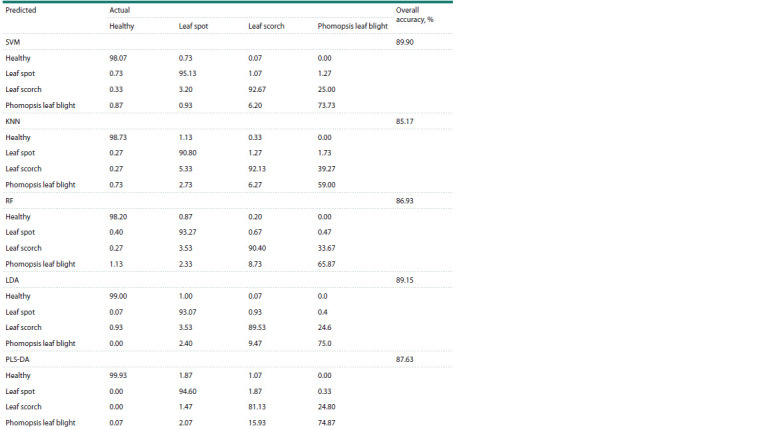
Confusion matrices for hyperspectral image classification by different methods using the full spectrum Notе. SVM – the support vector machine method; KNN – the K-nearest neighbors method; LDA – the linear discriminant analysis method; PLS-DA – the partial least squares discriminant analysis method; RF – the random forest method.

The support vector machine method on the full range of wavelengths demonstrated the highest classification accuracy (90 %), while the K-nearest neighbors method showed the lowest accuracy (85 %).

Classification results based on sets of optimal wavelengths and vegetation indices

Each of the five classification models (SVM, KNN, LDA, PLS-DA, RF) was trained on sets of optimal wavelengths obtained by applying four different dimensionality reduction methods (ROC curve analysis, derivative analysis, PLS-DA, ReliefF), as well as on a set of 16 vegetation indices (Table 3).

**Table 3.**
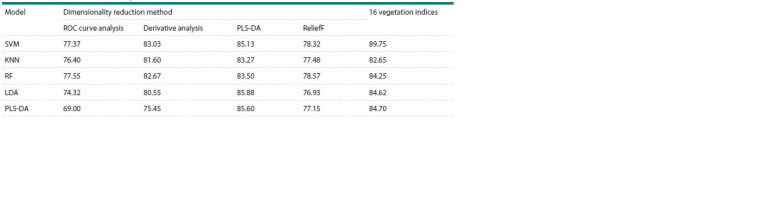
Overall classification accuracy (%) for different models using selected wavelengths and vegetation indices Notе. SVM – the support vector machine method; KNN – the K-nearest neighbors method; RF – the random forest method; LDA – the linear discriminant analysis method; PLS-DA – the partial least squares discriminant analysis method.

As can be seen from the results presented in Table 3, the overall classification accuracy of all methods decreased compared to the classification using the full spectrum. The highest classification accuracy for all models was obtained for the set of vegetation indices and for the set of wavelengths selected by the PLS-DA method.

Identification of fungal diseases of strawberry

**Fig. 4.**
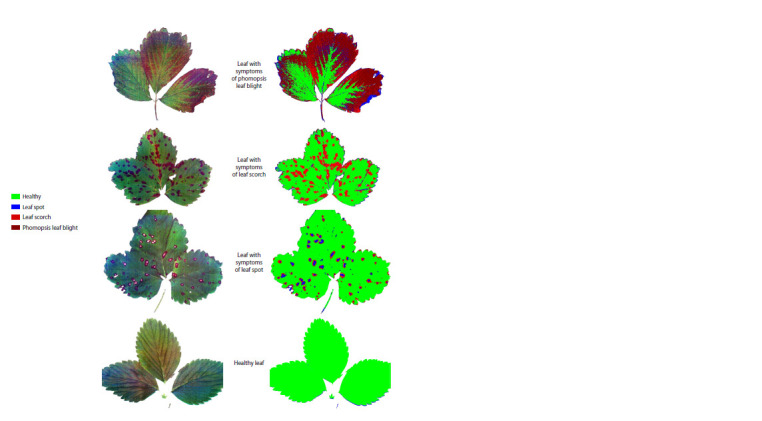
Visualization of strawberry disease classification using support vector machines on a full range of values. The left column shows the original color images of the strawberry leaves and the right column visualizes the classification results after applying the SVM model to the full spectrum in every single pixel in the images.

Trained and optimized models can be used to detect and differentiate fungal diseases of strawberry. Figure 4 shows an example of the application of the SVM model for the diagnosis of different types of fungal diseases.

## Discussion

The analysis of hyperspectral images by machine learning methods has already been successfully applied in scientific research to detect strawberry diseases. For example, G. Wu et al. (2023) focused on the potential of using hyperspectral imaging (HSI) combined with spectral features, vegetation indices (VIs), and textural features (TFs) for the detection of gray mold on strawberry leaves under laboratory conditions. Three machine learning models (ELM, KNN, SVM) were trained and optimized. The overall classification accuracy of the models reached 96 %.

In (Jiang et al., 2021) six machine learning methods (SVM, ELM, KNN, PLS-DA, RF, NB) were developed based on the selected spectral fingerprint features for early identification of anthracnose and gray mold in strawberries using a hyperspectral imaging system. Most classification models obtain relatively good accuracy (100 %) and robust performance, recognizing asymptomatic fungus infections classes before the obvious signs of disease appear notably in the strawberry. In our study, the obtained accuracy of disease classification did not exceed 90 %. This result can be explained by several reasons. First, three types of disease were considered at once, rather than one or two as in other studies. Secondly, successful differentiation of diseases requires a difference in the spectral characteristics of plant leaves affected by pathogens. Our study revealed that the main errors in classification occur when differentiating leaf scorch and phomopsis leaf blight, since these diseases have a similar reflectance spectrum. A possible way to improve classification accuracy is to use convolutional neural networks that take into account not only spectral but also textural characteristics of the affected leaves, such as shape and location of spots.

The choice of classification method depends on the diseases under study. Among the five popular machine learning models we considered (SVM, KNN, LDA, PLS-DA, RF), the support vector machine (SVM) demonstrated the best classification accuracy, which is in agreement with the results of other studies (Benos et al., 2021).

In order to reduce dimensionality and select optimal wavelengths for model building, researchers have applied various methods. Thus, the CARS, CARS-RF, ReliefF, and ROC algorithms were used in (Luo et al., 2012; Jiang et al., 2021; Wu et al., 2023). In many studies, dimensionality reduction does not reduce the accuracy of the models, but in our case, all wavelengths were significant and the classification accuracy decreased slightly compared to the full spectrum

The obtained results of laboratory studies indicate the potential of using hyperspectral imaging methods for diagnosing fungal diseases of strawberries in agricultural production. Scientific publications have already described examples of successful application of hyperspectral sensors mounted on UAVs for diagnostics of biotic and abiotic plant stresses (Yang et al., 2017).

In our further research, we plan to test the application of hyperspectral imaging methods in field conditions to automate the diagnosis of fungal diseases of strawberries.

## Conclusion

This study explored the feasibility of using hyperspectral imaging technique combined with machine learning for the detection and identification of leaf spot, leaf scorch and phomopsis leaf blight diseases on strawberry leaves in the presence of visible symptoms. In order to identify the strawberry leaves disease effectively, diverse classifiers (SVM, KNN, LDA, PLS-DA, RF) were developed and evaluated using the full spectrum. The Support Vector Machine (SVM) demonstrated the highest classification accuracy of 89.9 % on 131 wavelengths in the range of 470–900 nm. In order to simplify the models and increase the speed of data processing, four different dimensionality reduction methods were considered (ROC curve analysis, derivative analysis, PLS- DA, ReliefF). Moreover, 16 vegetation indices were used as features. The overall classification accuracy of all methods decreased slightly compared to classification using the full spectrum. The set of 16 optimal wavelengths obtained by the PLS-DA method and the set of 16 vegetation indices had higher classification accuracy than the other wavelength sets.

## Conflict of interest

The authors declare no conflict of interest.
